# Characteristics, sources and risk assessments of heavy metal pollution in soils of typical chlor-alkali residue storage sites in northeastern China

**DOI:** 10.1371/journal.pone.0273434

**Published:** 2022-09-09

**Authors:** Zhiyuan Wu, Dan Zhang, Tianxiang Xia, Xiaoyang Jia

**Affiliations:** Beijing Municipal Research Institute of Eco-Environmental Protection, National Engineering Research Center of Urban Environmental Pollution Control, Beijing Key Laboratory for Risk Modeling and Remediation of Contaminated Sites, Beijing, China; Gifu University, JAPAN

## Abstract

In this study, thirty-four soil samples from a typical chlor-alkali slag residue storage site near the city of Qiqihar in northeastern China were collected and their arsenic, cadmium, chromium, copper, mercury, nickel, lead and zinc concentrations were determined. Sources of these heavy metals were analyzed with a positive matrix factorization model, and the health risks associated with different pollution sources were calculated. The results showed that mercury was the main heavy metal pollutant at the site (maximum concentration of 112.19 mg.kg^−1^) and the soil was also contaminated with arsenic, copper and lead. The sources of eight heavy metals were: mixed oil refinery wastewater and parent material (arsenic, chromium, copper and lead), vinyl chloride waste source (mercury), parent material (cadmium, nickel and zinc). The average potential ecological risk of the soil was 22344.39, with vinyl chloride waste source contributing 99.85% of this risk. The average carcinogenic risk of a mixture of oil refinery wastewater and parent material for children and adults was 9.06×10^−6^ and 6.36×10^−6^, respectively, accounting for 99.9% (children) and 99.48% (adults) of the total average carcinogenic risk. The average hazard index of vinyl chloride waste source for children and adults was 0.6 and 0.38, respectively, which accounted for 64.13% (children) and 52.34% (adults) of the total hazard index. These results provide a reference for soil pollution risk assessments at this type of site.

## Introduction

With an increased understanding regarding the importance of soil environments, treating industrial pollution sites has become an urgent research topic for soil restoration. The evaluation and restoration of sites polluted with heavy metals (HMs) is an important part of treating industrial pollution. Soil HM pollution is generally long-lasting and stable. Studies have shown that cadmium (Cd) can remain in soil for 75 to 380 yrs, mercury (Hg) for 500 to 1000 yrs, and nickel (Ni), copper (Cu), lead (Pb) and zinc (Zn) for 1000 to 3000 yrs [1]. Excess HMs in soil destroy the ecological environment and they can also migrate to other environmental settings (e.g., atmosphere and water bodies) and endanger animal and human health through the food chain, breathing, drinking water and skin contact [2]. Specifically, excess Pb can cause damage to organs such as the human reproductive and immune systems and kidneys [3], and excess Cd can lead to loss of bone density, kidney damage and cancer [4]. Therefore, it is very important to: investigate soil HMs at industrial pollution sites; systematically evaluate the degree of soil HM pollution; determine the health risk to the population.

Unlike other types of land (e.g., farmland), HMs in soils at industrial pollution sites are closely linked to industrial production activities. In the northern estuary area of Liaodong Bay (China), Cu, Zn and Pb are mainly derived from natural sources and chromium (Cr) and Ni mainly originate from domestic and industrial wastewater discharge. Phosphorus-based fertilizer, petrochemical production and industrial activities are the main anthropogenic sources [5]. Zn found in soils around waste incineration sites mainly comes from the incineration plants. Similarly, Pb, Cu and Cd are mostly produced by waste incineration plants, but can also be contributed by natural sources. Cr and Ni are mainly derived from natural sources. Most Hg originates from waste incineration plants and coal-fired emissions, and arsenic (As) is mainly derived from specific industrial pollution [6]. In Shou steel plant and the surrounding soil, Ni and As are mainly derived from soil-based metals, whereas Cu, Zn, Pb and Cd are mainly derived from industrial smelting and vehicle emissions. Cr content is controlled by soil-based metals and anthropogenic pollution [7]. Pb, Cd, Cu, Zn and As levels at a steel plant in western Fujian Province are mainly affected by pollutants emitted by steel mills, and the Cr and Ni levels are mainly affected by natural sources [8]. The contributions of five pollution sources in an eastern open-pit mining area were 20.79%, 16.83%, 16.83%, 27.72% and 17.82% [9]. Cu, Cr, Pb and Zn in Shou steel are derived from steel smelting, while Cd and Sb are derived from vehicle emissions and Hg is released from coal combustion [10].

Utilizing the precise analysis of HM sources, scholars have evaluated the ecological and human health risks of soil HMs from pollution. In Silver City (China), coal combustion and related activities are the most significant sources of soil HMs and influence the carcinogenic risk (CR) and hazard index (HI) of the region. The use of pollution sources for human health risk assessments is more meaningful than only using CR and/or HI thresholds [11]. For example, in a former Chinese electronic waste dismantling center, parent materials from the plant, fertilizer application, industrial discharge and vehicle emissions accounted for 52.9%, 19.0% and 28.1%, respectively, of the total HI and 39.2%, 45.3% and 15.5%, respectively, of the total cancer risk [12]. The main HMs in farmland soil of the Kowloon River basin are from natural sources, agricultural activities, coal-fired release and industrial activities; their combined contribution to HM concentrations in these soils was 37.0%, 26.7%, 17.6% and 18.7%, respectively [13]. In addition, using pollution sources in human health risk assessments is also important for guidance to similar business and production activities [10].

**Fig 1:**
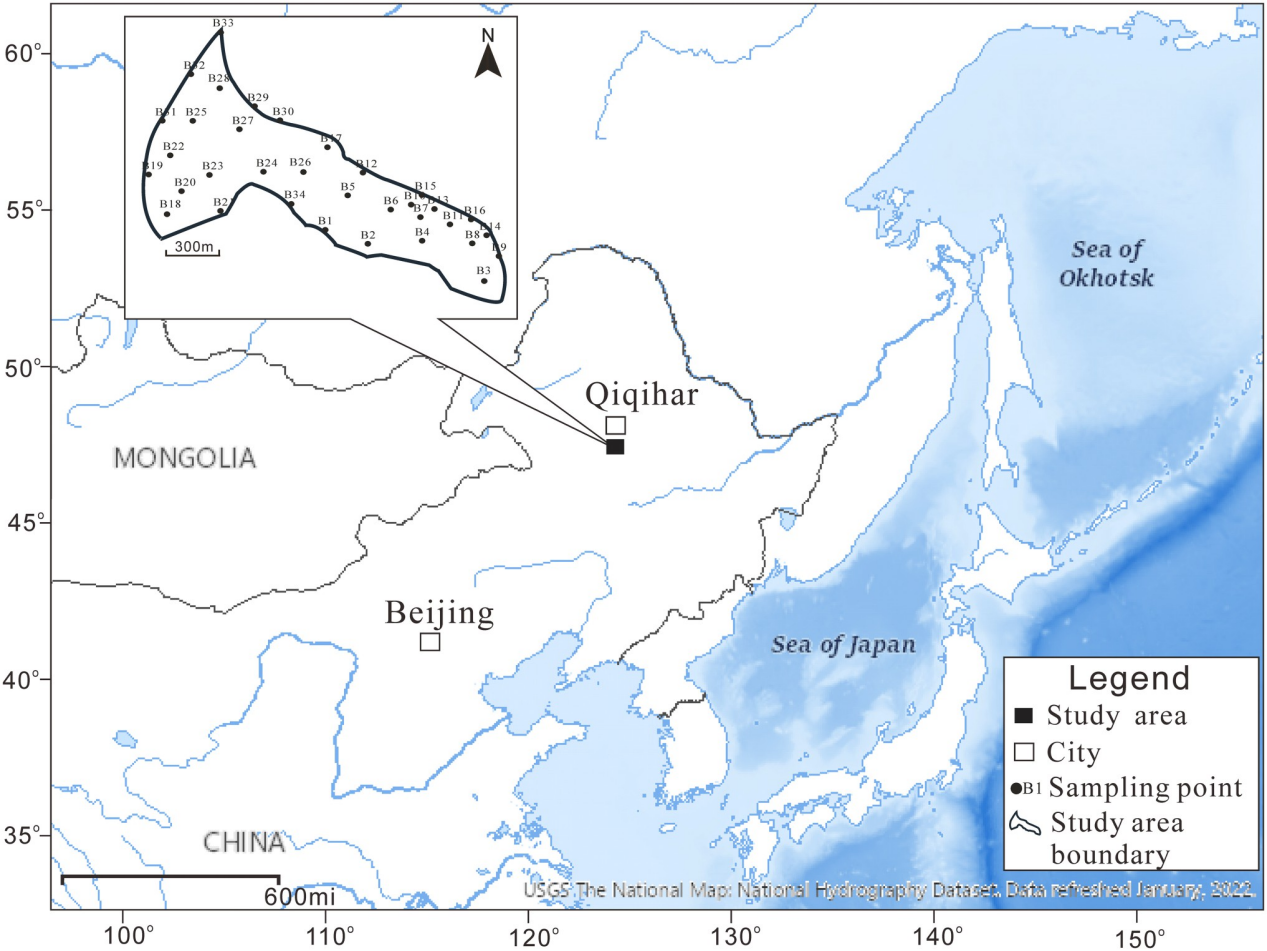
Site location and sampling layout.

Qiqihar, an old industrial city in China, has had a large number of chlor-alkali enterprises built in the area, which generated a large amount of Hg-containing waste slag. In this study, a typical Hg-containing waste slag stockpile site (47°08′46.98” N, 123°53’53.70” E), 22 km away from Qiqihar (Fig 1), was selected. The total study area is approximately 28.57×10^4^ m^2^. There have been active industrial sites in the study area since the 1950s and large amounts of production waste have been discharged in the form of wastewater and residue. The degree and sources of soil HM pollution in these slag sites were analyzed, and the ecological and human health risks were calculated based on the pollution sources. The aims of the study were to: understand soil contamination at the site; analyze whether pollution at the site spreads outwards; identify the ecological and human risks.

## Materials and methods

### Study area and sample collection

The area surrounding the study site contains production facilities for caustic soda, polyvinyl chloride (PVC) and oil refining. The surface is covered by calcium carbide slag (0.3–4 m thick). This study analyzed soil HM pollution under the calcium carbide slag. Soil that was 0.5 m below the calcium carbide slag was collected for analysis using the judgement method [14]. In total, 34 sampling points (B1–B34) were defined in the study area (solid black circle in Fig 1), with one sample taken from each sampling point and one parallel sample set for 10 soil samples. Soil samples were collected between May and June 2019 with the permission and assistance of the Qiqihar Environmental Protection Bureau.

### Sample preparation and heavy metal measurement

The soil samples were dried naturally and reduced to 100 g by quadratic fractionation. Foreign matter, such as stones and plant residue, was then removed and the samples ground in an agate mortar before being passed through nylon sieve with aperture of 0.1mm.

The Cu, Zn, Cr and Ni concentrations were measured by flame atomic absorption spectrophotometry. For each sample, 0.1 g of soil was placed in the digestion tank of a MD7 microwave digester. Then, 4 mL of concentrated nitric acid was added, and the mixture digested for 4 min at 150°C and 1.5 MPa and then for 4 min at 180°C and 2.0 MPa. At the end of digestion, the digestion tank was removed and allowed to cool until the lid could be easily unscrewed. The digestion tank was opened in a fume hood. The solution was removed and transferred to a 100 mL volumetric flask, filled to volume with 2% nitric acid and shaken well. Finally, 25 mL of the solution was placed in a colorimetric tube and the absorbance measured with an AA-6300C flame atomic absorption spectrophotometer. The regression equation of the standard curve was used to calculate the Cu, Zn, Cr and Ni contents.

Pb and Cd concentrations were measured with graphite furnace atomic absorption spectrophotometry. For each sample, 0.20 g of soil was placed into a microwave digestion tank with a few drops of water, 8 mL of nitric acid, 4 mL of hydrofluoric acid and 4 mL of hydrochloric acid. The sample and solution were covered with an inner lid and an explosion-proof membrane, placed in a MD7 Microwave Digester and heated. Microwave digestion in a HNO_3_–HF–HCl solution completely destroys the mineral lattice in the soil and puts all of the elements into solution. After digestion, the solution was cooled and transferred to a 50 mL volumetric flask. The sample was then injected into a graphite furnace at high temperature to dissociate Pb and Cd into their ground-state atoms. The atomic vapor selectively absorbs characteristic spectral lines at 283.3 nm and 228.8 nm emitted by a sharp-line light source (Pb and Cd hollow cathode lamp). The soil Pb and Cd content was determined according to the absorbance.

As and Hg concentrations were determined by the atomic fluorescence method. For each sample, 0.30 g of soil and 5 mL of water were weighed and put it into a 25 mL coloration tube. It was covered with a breathable glass plug, left overnight and then digested for 2 h in a boiling water bath (shaken once every 30 min). After removing the solution and cooling, it was left to stand overnight. The next day, 5 mL of supernatant was taken and 3 mL of 5% thiourea with 5% ascorbic acid were added, then shaken. Subsequently, 2 mL of concentrated hydrochloric acid was added to fix the volume at 10 mL, and the solution was left for 30–60 min. As and Hg were determined with an AFS-2300 atomic fluorescence spectrophotometer.

To ensure the accuracy and precision of the experimental results, reagent blanks were used to reduce errors. The relative standard deviation was controlled at ≤5% and the recoveries were 80%–120% [15].

The pH values were measuring with the potentiometric method under a 2.5:1 soil:water mixture. Organic matter content was determined through potassium dichromate oxidation and total soil nitrogen by the semi-micro Kjeldahl method. Total phosphorus was determined by perchloric acid–sulfuric acid digestion. Total soil potassium was measured using the sodium hydroxide fusion–flame photometric method [16]. Soil cation exchange was determined via ammonium acetate exchange [17].

### Positive matrix factorization

Positive matrix factorization (PMF) relies on a receptor model that quantifies the contributions of sources to samples based on source composition or a fingerprint. The model has been used extensively to analyze HM sources in soils [18–20]. In this study, PMF version 5.0 (U.S. EPA, Washington, DC) was used for source apportionment. This model decomposes the original matrix *x*_*ij*_ into two factor matrices, *g*_*ik*_ and *f*_*jk*_, and a residual error matrix e_*ij*_. The basic equation is:

$$x_{ij}=\sum_{k=1}^{p}g_{ik}\cdot f_{kj}+e_{ij}$$ (1)

where *x*_*ij*_ is the content of HM *j*_*th*_ in sample *i*, *g*_*ik*_ is the contribution of source *k*_*th*_ for sample *i*, and *f*_*kj*_ is the source profile of *j*_*th*_ in source *k*. The residual error matrix, *e*_*ij*_, is calculated as the minimum value of objective function Q:

$$Q=\sum_{i=1}^{n}\sum_{j=1}^{m}{\left(\frac{e_{ij}}{u_{\text{ij}}}\right)}^{2}$$ (2)

where *u*_*ij*_ refers to the uncertainty of HM *j*_*th*_ in *i* number of samples. The main feature of PMF is that the model requires HM sample contents and uncertainties, which are used to analyze the quality of the content values individually. The uncertainties can be calculated through various methods [21]. In this study, the uncertainties were calculated as:

$$\text{If}\;c\;\leq \text{MDL},\;u_{ij}=\frac{5}{6}\times \text{MDL}$$ (3)

$$\text{If}\;c\;>\text{MDL},\;u_{ij}=\frac{1}{10}\times c$$ (4)

where *c* is the element content and MDL is the method detection limit.

### Statistical analysis

Descriptive and multivariate statistical analyses were used to analyze the sample data. Descriptive statistical analysis included maximum, minimum, average and median values, skewness, kurtosis and coefficient of variation. Different software programs were used for statistical analyses (SPSS 21.0), pollution source analysis (EPA PMF 5.0) and pollution distribution and mapping (ArcGIS 10.6).

### Ecological risk assessment

The calculation for a single pollution index is [22]:

$$P_{i}=C_{i}/S_{i}$$ (5)

where *Pi* is the single pollution index of HM *i*, *C*_*i*_ is the measured content of *i* in soil (mg kg^−1^), and *Si* is the environmental standard value of *i* in soil (mg kg^−1^). In this study, the second-level environmental quality standard values of soil HM pollutants in the *Soil Environmental Quality Standard* (GB15618-2008) were used to evaluate the soil HM pollution. Usually, *Pi* <1 indicates that the soil is not polluted, 1 ≤ *Pi*< 2 indicates that the soil is slightly polluted, 2 ≤ *Pi*< 3 indicates that the soil is moderately polluted, and *Pi* ≥3 indicates that the soil is heavily polluted by HMs.

The ecological risk assessment is based on a set of methods established by Hakanson. It utilizes sedimentology to evaluate the potential ecological hazards of HMs based on their properties and environmental characteristics [23]. The potential ecological risk factor $E_{r}^{i}$ of a single HM is calculated as:

$$E_{r}^{i}=T_{r}^{i}\times \frac{C^{i}}{C_{n}^{i}}$$ (6)

where *C*^*i*^ is the content of HM *i*, $C_{n}^{i}$ is the reference value of *i*, based on the arithmetic mean of the Heilongjiang Province soil background value [24], and $T_{r}^{i}$ is the toxicity response coefficient of a HM, which reflects the toxicity of a HM and the sensitivity of the soil to HM pollution. The corresponding toxicity coefficients for As, Cd, Cr, Cu, Hg, Ni, Pb and Zn are 10, 30, 2, 5, 40, 5, 5 and 1, respectively [25, 26].

The comprehensive potential ecological risk index (RI; Table 1) of HMs is expressed as the sum of the $E_{r}^{i}$ value of each HM:

$$\text{RI}=\sum_{i=1}^{m}E_{r}^{i}$$ (7)

**Table 1:** Classification of the Hakanson potential ecological risk.

Single ecological risk factor (${\text{E}}_{\text{r}}^{\text{i}}$)		Comprehensive ecological risk index (RI)	
Level	Score	Level	Score
**Low ecological risk**	<40	Low ecological risk	<150
**Medium ecological risk**	40–80	Medium ecological risk	150–300
**Higher ecological risk**	80–160	High ecological risk	300–600
**High ecological risk**	160–320	Extremely high ecological risk	>600

### Human health risk assessment

The human health risk assessment was carried out according to the methods specified in the *Technical Guidelines for Risk Assessment of Contaminated Sites* (HJ 25.3–2019). The second land use category was chosen as the exposure scenario, and the sensitive receptor was “adult”. The contribution to the overall human health risk of each pollution source was quantitatively examined with PMF. The quantitative analysis expressions are shown in Eqs (8) through (17) [11], and the parameters for each equation can be found in the technical guideline (HJ 25.3–2019). Due to the limited volatility of HMs, only oral ingestion, dermal contact and respiratory inhalation are considered main exposure routes. S1 Table shows the non-carcinogenic reference doses and carcinogenic slope factors of the three exposure pathways for each pollutant. S2 Table shows the relevant parameter values used in the human health risk assessments.

$$\text{Con}{\left({\text{CR}}_{ois}\right)}_{ij}={\text{Con}}_{ij}\times SF_{0}\times \frac{ABS_{0}\times \left(\frac{OISER_{a}\times ED_{a}\times EF_{a}}{BW_{a}}\right)}{AT_{ca}}\times 10^{-6}$$ (8)

$$\text{Con}{\left({\text{HI}}_{ois}\right)}_{ij}=\frac{Con_{ij}}{RfD_{0}\times SAF}\times \frac{ABS_{0}\times OSTR_{c}\times ED_{c}\times EF_{c}}{BW_{c}\times AT_{nc}}\times 10^{-6}$$ (9)

$$\text{Con}{\left({\text{CR}}_{dsc}\right)}_{ij}=Con_{ij}\times \frac{SF_{0}}{ABS_{gi}}\times \frac{ABS_{d}\times E_{v}\times \left(\frac{SAF_{a}\times SSAR_{a}\times ED_{a}\times EF_{a}}{BW_{a}}\right)}{AT_{ca}}\times 10^{-6}$$ (10)

$$\text{Con}{\left({\text{HI}}_{dsc}\right)}_{ij}=\frac{Con_{ij}}{RfD_{0}\times ABS_{gi}\times SAF}\times \frac{ABS_{d}\times E_{v}\times SAE_{c}\times SSAR_{c}\times ED_{c}\times EF_{c}}{AT_{nc}\times BW_{c}}\times 10^{-6}$$ (11)

$$\text{Con}{\left(CR_{pis}\right)}_{ij}={\text{Con}}_{ij}\times \frac{IUR\times BW_{a}}{DAIR_{a}}\times {\text{PM}}_{10}\times \;\;\frac{DAIR_{a}\times PLAF\times ED_{a}\times \left(fspo\times EFO_{a}\times +fspi\times EFI_{a}\right)}{BW_{a}\times AT_{ca}}\times 10^{-6}$$ (12)

$$\text{Con}{\left({\text{HI}}_{pis}\right)}_{ij}=\frac{Con_{ij}}{\frac{RfC\times DAIR_{a}}{BW_{a}}\times SAF}\times \frac{PM_{10}\times DAIR_{c}\times PLAF\times ED_{c}\times \left(fspo\times EFO_{c}\times +fspi\times EFI_{c}\right)}{BW_{c}\times AT_{nc}}\times 10^{-6}$$ (13)

$$\text{Con}{\left({\text{CR}}_{k}\right)}_{ij}=\text{Con}{\left({\text{CR}}_{ois}\right)}_{ij}+\text{Con}{\left({\text{CR}}_{dsc}\right)}_{ij}+\text{Con}{\left({\text{CR}}_{pis}\right)}_{ij}$$ (14)

$$\text{Con}{\left({\text{HI}}_{k}\right)}_{ij}=\text{Con}{\left({\text{HI}}_{ois}\right)}_{ij}+\text{Con}{\left({\text{HI}}_{dsc}\right)}_{ij}+\text{Con}{\left({\text{HI}}_{pis}\right)}_{ij}$$ (15)

$$\text{Con}{\left(\text{Total}-{\text{CR}}_{k}\right)}_{ij}=\sum \text{Con}{\left({\text{CR}}_{k}\right)}_{ij}$$ (16)

$$\text{Con}{\left(\text{Total}-{\text{HI}}_{k}\right)}_{ij}=\sum \text{Con}{\left({\text{HI}}_{k}\right)}_{ij}$$ (17)

where Con_*ij*_ is the pollutant content of i*th* element and j*th* source; Con(CR_*ois*_)_*ij*_ is the oral carcinogenic risk of i*th* element and j*th* source; Con(HI_*ois*_)_*ij*_ is the non-carcinogenic risk of oral ingestion of i*th* element and j*th* source; Con(CR_*des*_)_*ij*_ is the carcinogenic risk of dermal contact from i*th* element and j*th* source; Con(HQ_*des*_)_ij_ is the i*th* element from j*th* source for dermal contact and non-carcinogenic risk; Con(CR_*pis*_)_*ij*_ is the carcinogenic risk of i*th* element from j*th* source for respiratory inhalation; Con(HQ_*pis*_)_*ij*_ is the non-carcinogenic risk of i*th* element from j*th* source for respiratory inhalation; Con(CR_*k*_)_*ij*_ is the carcinogenic risk of i*th* element and j*th* source via all three pathways; Con(HI_*k*_)_*ij*_ is the non-carcinogenic risk from the third pathway of i*th* element and j*th* source; Con(total-CR_*k*_)_*ij*_ is the total carcinogenic risk of i*th* element from j*th* source; Con(total-HI_*k*_)_*ij*_ is the total non-carcinogenic risk of i*th* element from j*th* source.

## Results and discussion

### Heavy metal concentrations in the study area

The statistical results for the HM contents in all 34 soil samples are shown in Table 2. The results showed that the maximum concentrations of As, Cu, Hg and Pb were 16.88, 29.20, 112.19 and 120.00 mg.kg^−1^, respectively, all of which exceed background values. The maximum concentrations of Cd, Cr, Ni and Zn were 0.06, 45.30, 20.60 and 54.50 mg.kg^−1^, respectively, which do not exceed local soil background values. This suggested that there was no contamination in these four HMs. Smaller HM concentration variation coefficients indicate smaller differences and less dispersion (i.e., more uniform distribution). In contrast, greater differences and more dispersion degree indicate more uneven distributions [23, 27]. The soil Hg variation coefficient was 2.73, with large concentration differences and high dispersion, indicating that Hg is unevenly distributed. Variation coefficients of the other seven HMs were relatively small (<1), which indicates an even distribution in the soil. Single-factor pollution indices of Hg and Pb were 5609.50 and 5.29, respectively, which indicated an association with heavy pollution. The single-factor pollution indices of As and Cu were 1.81 and 1.56, respectively, suggesting slight pollution. In contrast, the single-factor pollution indices of Cd (0.75), Cr (0.89), Ni (0.85) and Zn (0.95) indicated that these four HMs were not associated with pollution. Thus, soil at the site was most seriously polluted by Hg.

**Table 2:** Descriptive statistics of soil heavy metal (HM) concentrations, pH and total organic content at the study site (*n* = 34).

Study site									Farmland	Background value	Screening value	Single factor pollution index *P*_*i*_
HMs	Average	Max	Min	Med	SD	CV	Skew	Kurt	Average			
**As (mg.kg** ^**-1**^ **)**	7.37	16.88	3.39	6.76	2.85	0.39	1.71	3.56	6.10	9.33	20	1.81
**Cd (mg.kg** ^**-1**^ **)**	0.03	0.06	0.01	0.03	0.01	0.39	0.29	-0.84	0.06	0.08	20	0.75
**Cr (mg.kg** ^**-1**^ **)**	26.98	45.30	8.99	26.70	9.40	0.35	0.11	-0.46	1.77	50.82	-	0.89
**Cu (mg.kg** ^**-1**^ **)**	11.72	29.20	3.72	10.16	5.28	0.45	1.41	2.88	11.50	18.74	2000	1.56
**Hg (mg.kg** ^**-1**^ **)**	8.92	112.19	0.01	0.07	24.33	2.73	3.23	10.57	0.13	0.02	8	5609.50
**Ni (mg.kg** ^**-1**^ **)**	11.78	20.60	1.61	11.50	4.41	0.37	0.22	0.11	10.69	24.16	150	0.85
**Pb (mg.kg** ^**-1**^ **)**	26.41	120.00	14.30	19.00	23.27	0.88	3.26	10.42	21.67	22.70	400	5.29
**Zn (mg.kg** ^**-1**^ **)**	32.57	54.50	10.50	32.00	9.59	0.29	0.30	0.43	-	57.34	-	0.95
**pH**	9.70	11.25	7.82	9.78	0.86	0.09	-0.20	-0.53	-	-	-	-
**Total organic matter (%)**	0.57	2.49	0.19	0.50	0.47	0.82	2.99	9.94	-	-	-	-

Note: - indicates no data.

**Fig 2:**
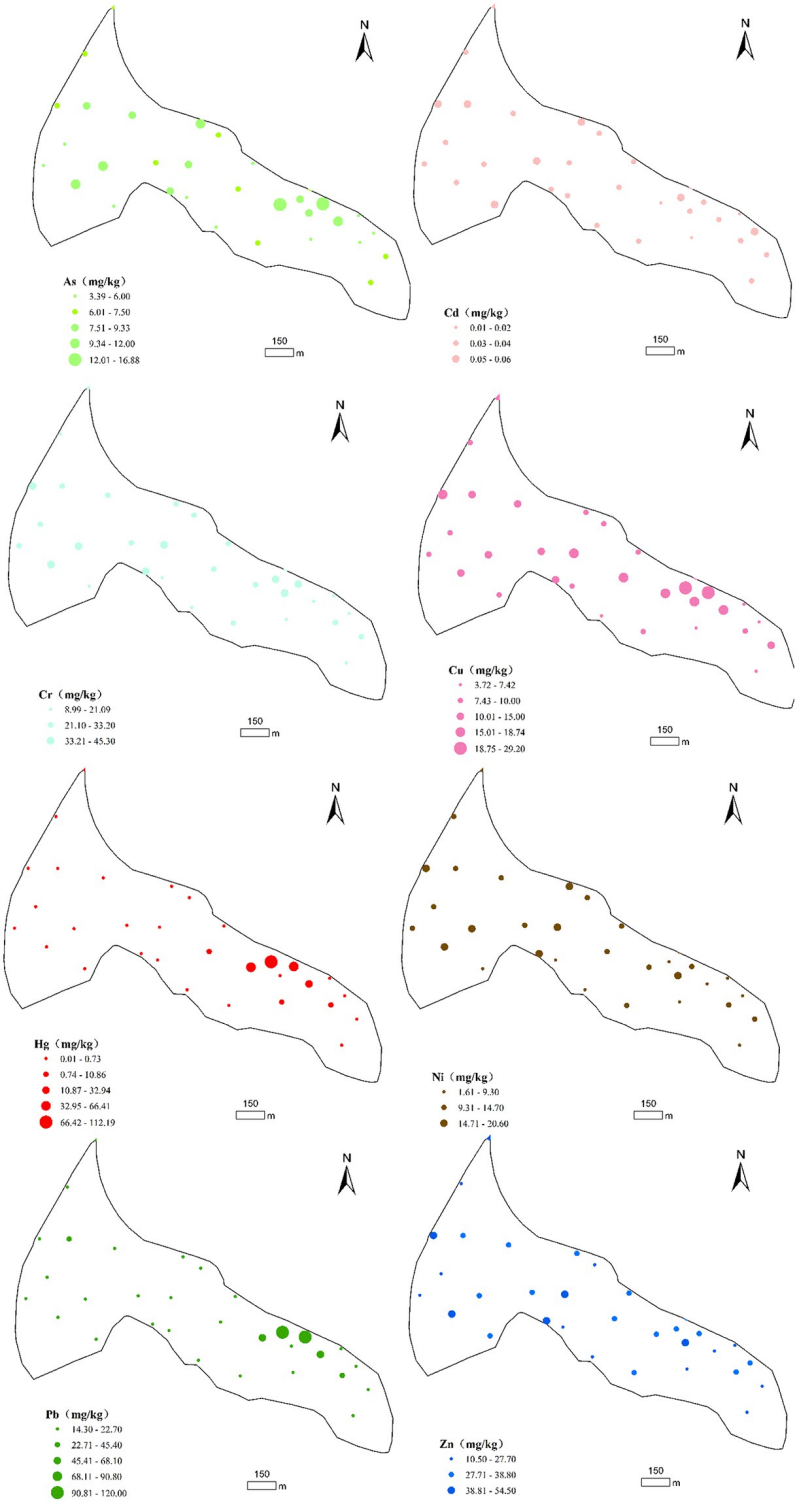
Concentrations of eight heavy metals, arsenic (As), cadmium (Cd), chromium (Cr), copper (Cu), nickel (Ni), mercury (Hg), lead (Pb) and zinc (Zn) at 34 sampling points within the study site.

The maximum soil concentrations of As, Cu, Hg and Pb are mainly distributed in the northeastern part of the site, where the terrain is low and there were large amounts of industrial wastewater discharge and stockpiling (Fig 2). It is therefore likely that the higher concentrations of HMs in this area are related to industrial wastewater discharge. Cd, Cr, Ni and Zn are more evenly distributed within the industrial site, which may be a reason why these HMs do not reflect contamination. Overall, the results suggest that the HM distribution is related to industrial effluent discharge from outside the site and is not directly related to industrial waste deposition.

The soil physical and chemical properties were also analyzed to better understand characteristics of the soil at the study site. The average soil organic matter content at the site was 0.57% (maximum value of 2.49%) so the organic matter content is low overall. The average soil pH value was 9.7, which indicates strongly alkaline soil. Higher pH values greatly reduce the availability of soil phosphorus, potassium and nitrogen [28]. Therefore, in addition to HMs, the soil in this area is affected by Hg-containing waste residues, and the physical and chemical properties of the soil have also been significantly affected.

### Heavy metal source analysis

#### Correlation analysis

**Fig 3:**
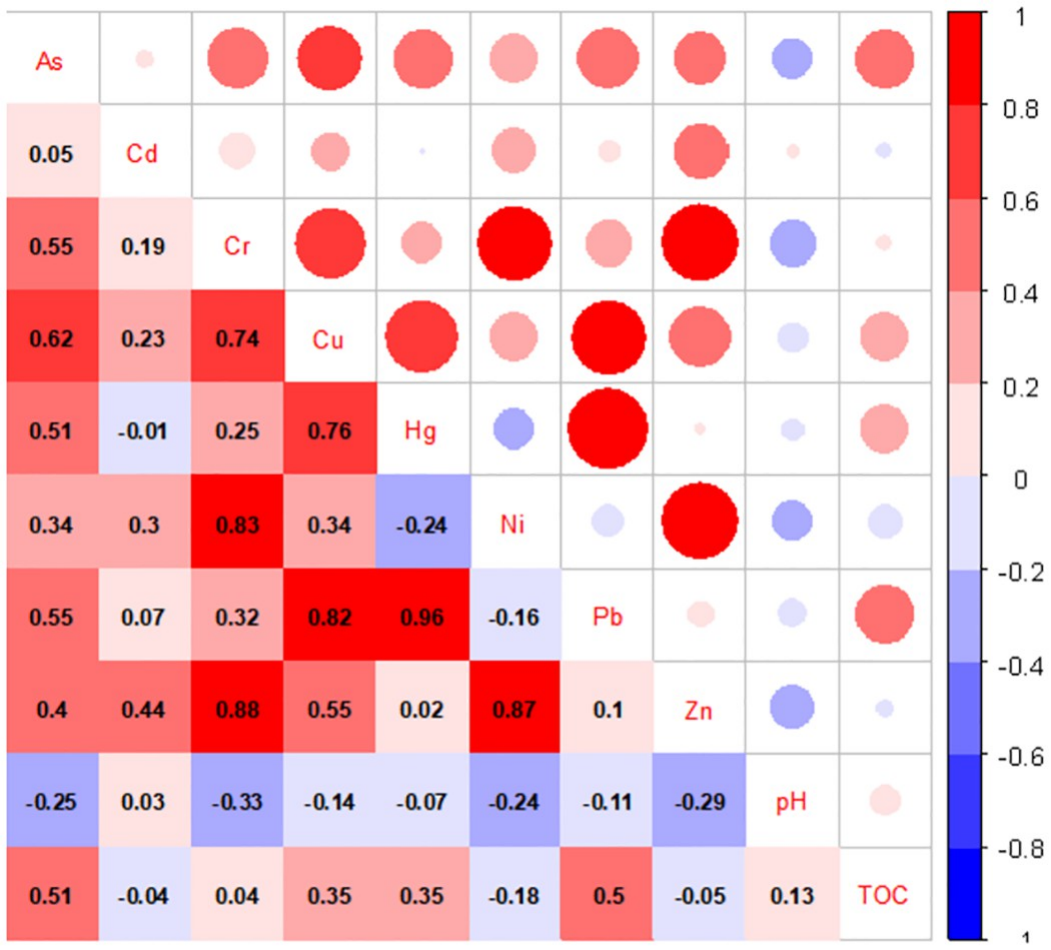
Relationships among soil HM concentrations, pH and total organic carbon (TOC).

The elemental correlations were significant or extremely significant, indicating homology or compound pollution among the elements [29, 30]. The Pearson correlation analysis (Fig 3) showed that Cu, Hg and Pb were significantly correlated (P <0.01), with correlation coefficients all >0.7, which indicates that these HMs have the same source. As was also significantly correlated (P <0.01) with Cu, Hg and Pb, but they had low correlation coefficients (<0.65); this indicates that As, Cu, Hg and Pb could have similar sources. Cd and Zn were significantly correlated (P <0.01), but exhibited a low correlation coefficient (<0.5) and there were also no correlations between Cd and the other six other HMs, which indicates that Cd has a different source. The soil HM concentrations, with the exception of Hg and Pb, were normally distributed, which indicates that Hg and Pb contamination may have been caused by anthropogenic sources [31].

#### Positive matrix factorization

**Fig 4:**
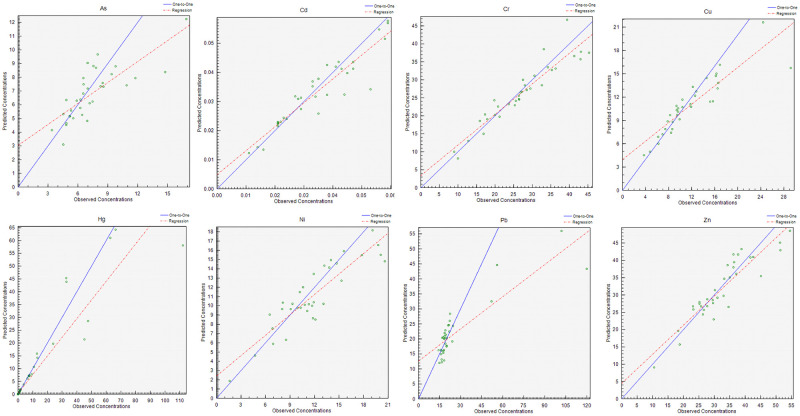
Comparison of observed heavy metal concentrations and predicted values from the positive matrix factorization model.

**Fig 5:**
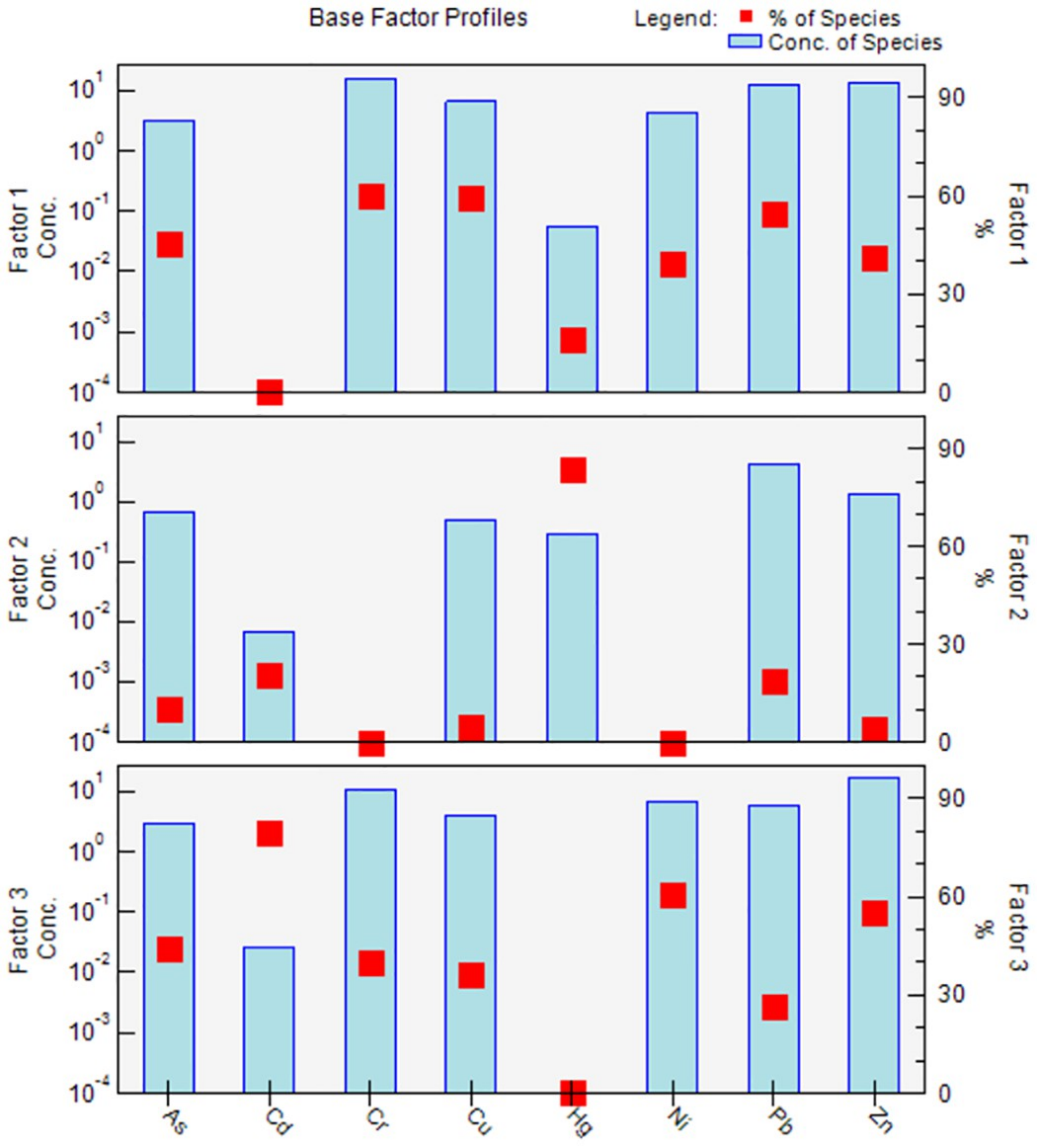
The contribution of different heavy metal sources based on positive matrix factorization.

PMF was used in this study to analyze the contributions of different pollution sources to soil HMs and identified three factors (after 20 iterations to obtain a low Q value of 21.5). All residual values were between −3 and 3 and the calculations were stable. The correlation coefficients between the predicted concentrations and measured HM concentrations obtained by the PMF model simulation were 0.83–0.95 (Fig 4), with a good fit. Based on reasoning and a low Q value (0.1), three main factors were selected (Fig 5). These results show that analytical values were consistent with simulation results [32].

As (45.5%), Cr (59.8%), Cu (59.3%) and Pb (54.6%) had higher loads on Factor 1. The average As, Cr, Cu and Pb concentrations did not exceed background values, which shows that these four HMs are mainly derived from parent material [33]. However, some of the As (six sampling points), Cu (two sampling points) and Pb (six sampling points) concentrations exceeded the local soil background values, which indicates that there is some degree of anthropogenic pollution in the soil. The analysis showed that higher As, Cu and Pb concentrations are mainly related to wastewater discharge in the northern industrial production area [34]. Large amounts of sulfide are generated during oil refining and then discharged into the site through sewage outlets. Therefore, Factor 1 represents a mixed source consisting of oil refinery wastewater and parent material.

Hg (83.8%) had a relatively high load on Factor 2. The Hg concentrations at twenty-nine sampling points exceeded the soil background value (0.02 mg kg^−1^). Among these, the soil Hg concentrations at six sampling points exceeded the screening value (8 mg kg^−1^), indicating anthropogenic pollution [35]. Activity in the northern industrial zone suggests that large amounts of Hg-containing waste were generated during PVC production [36, 37]. This Hg-containing waste was discharged along with wastewater, causing Hg pollution at the study site. Therefore, Factor 2 represents vinyl chloride waste source.

Cd (79.5%), Ni (61.1%) and Zn (55%) had higher loads on Factor 3. The maximum values of these HMs did not exceed local soil background values, which indicates that they are mainly derived from parent material [38]. Therefore, Factor 3 represents a parent material source.

**Fig 6:**
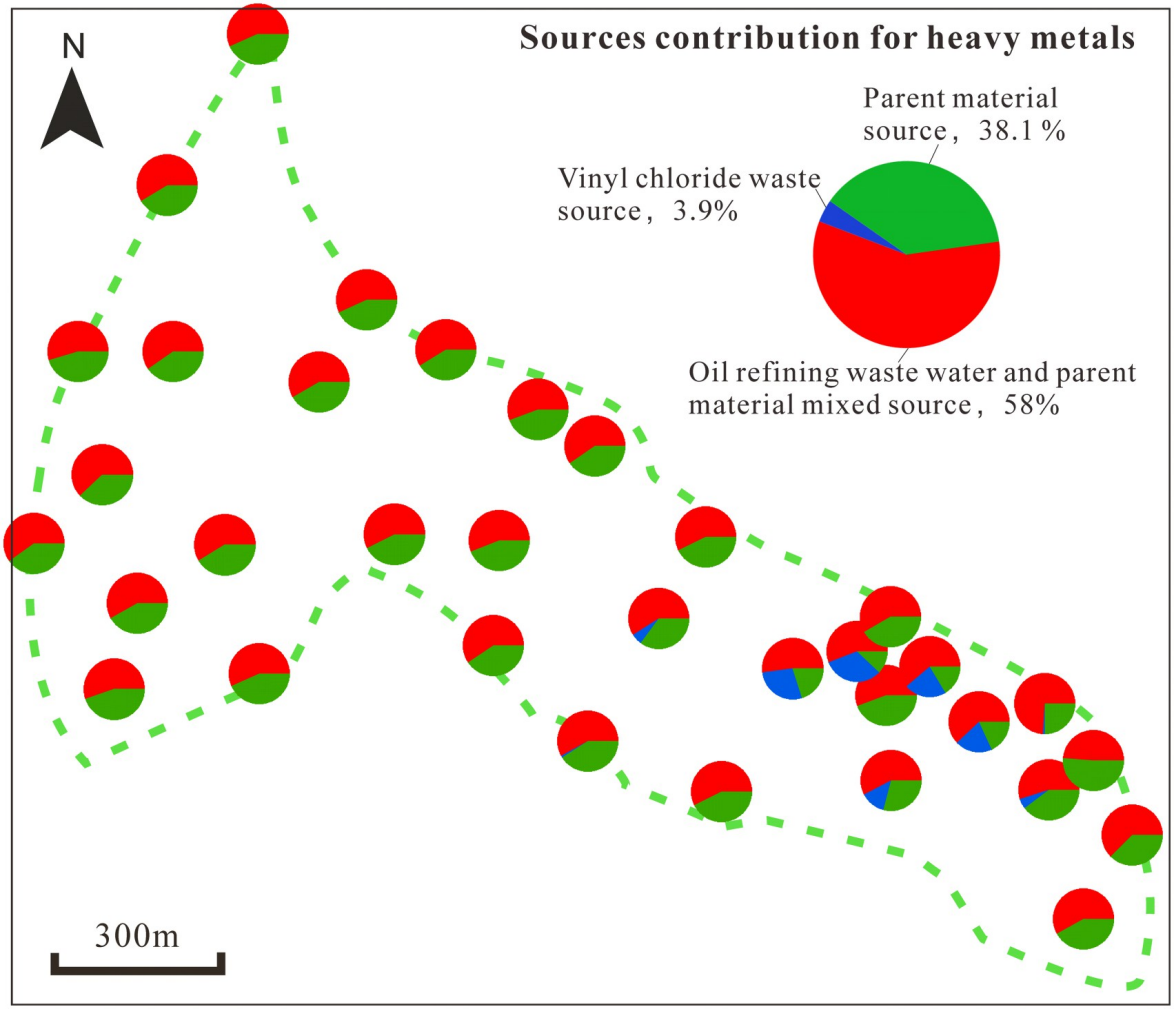
Spatial distribution of heavy metal sources.

These sources (oil refinery wastewater and parent material, vinyl chloride waste, and parent material) were identified by analyzing eight HMs in soils from 34 sampling points using PMF (Fig 6). The proportion of these three pollution sources to the total HM concentrations were 58%, 3.9% and 38.1%, respectively. Thus, soil HM contamination at the site is significantly affected by industrial production processes and demonstrates point source pollution.

### Ecological risk

**Fig 7:**
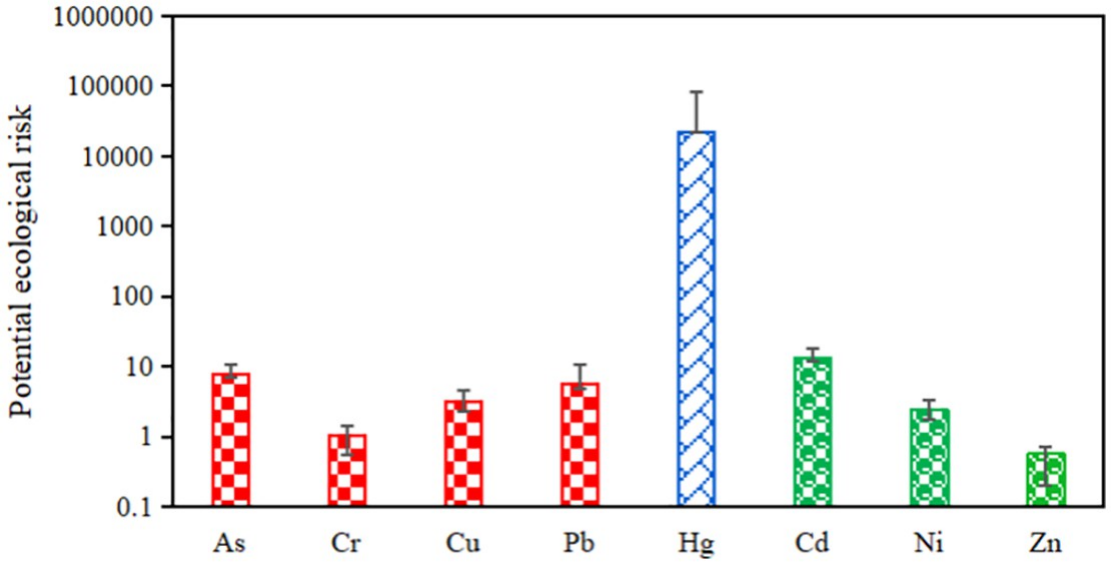
Potential ecological risks of eight heavy metals in the soil of the study site.

**Fig 8:**
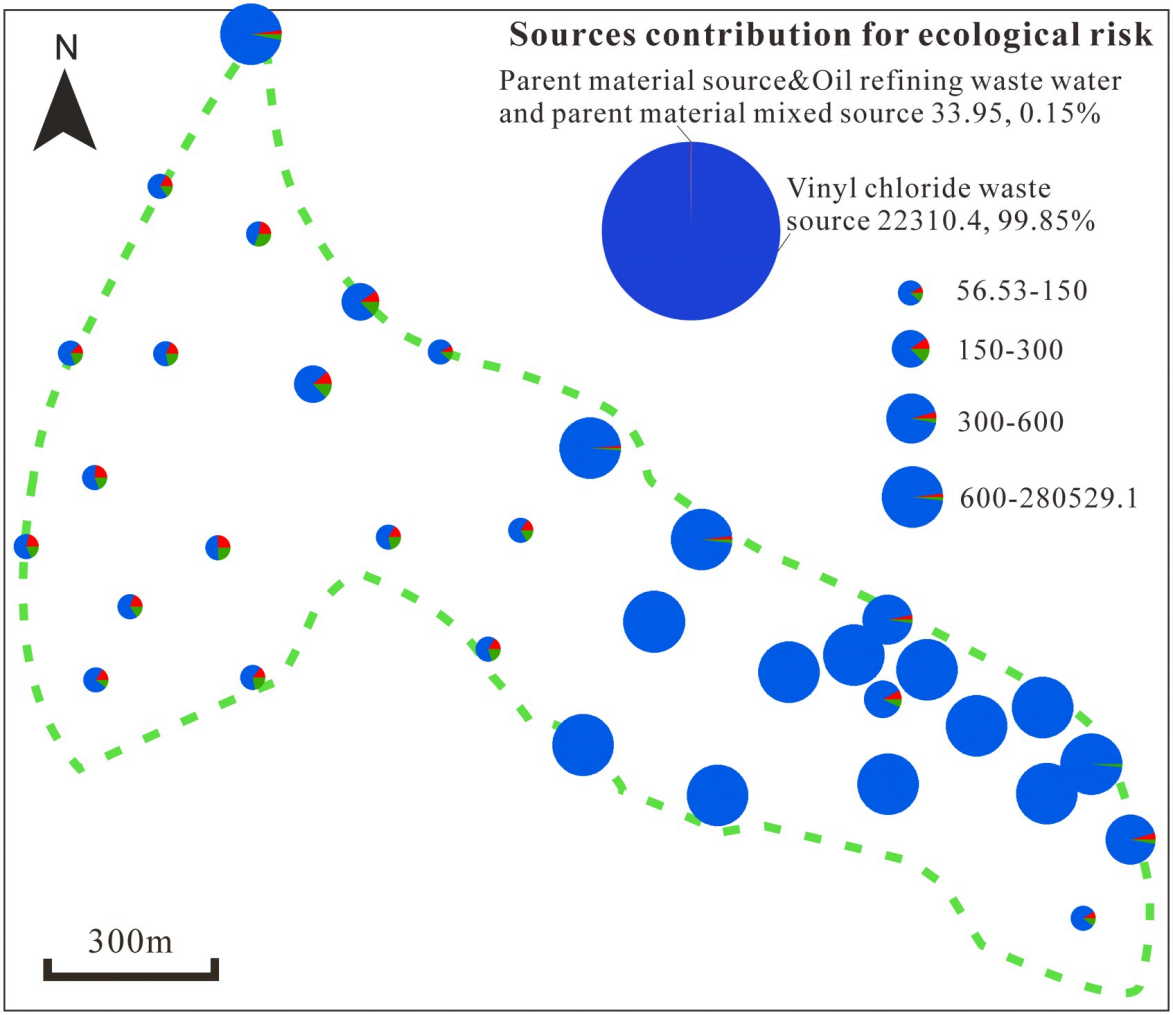
The contribution of heavy metal sources to the ecological risk.

The Hakanson potential ecological risk index method was used to measure the ecological risk of HMs contained in soil at the study site. The RI considers soil HM concentrations and links the ecological and environmental effects and HM toxicology, which comprehensively reflects the degree of HM stress on the ecological environment [39, 40]. The average potential ecological risk factors of As, Cd, Cr, Cu, Ni, Hg, Pb and Zn were 3.05, 0.37, 1.41, 5.13, 60818, 5.14, 0.91 and 0.17, respectively (Fig 7). Except for Hg, the ecological risks of the HMs appear low. However, the analysis showed that the ecological risks of the HMs in all samples ranged from 56.54 to 280529.1 (average of 22344.39), which suggests extremely high ecological risk. The samples with extremely high ecological risks are mainly located in the eastern part of the study area. The contribution of vinyl chloride waste source to the ecological risk reached 99.85% and was the main cause of the high ecological risk of the soil (Fig 8). The combined contribution of the other two HM sources to the total ecological risk was low (0.15%).

### Human health risk assessments of the different groups

#### Deterministic risk assessment

The reference dose and slope factor of each HMs are shown in S1 Table. According to the *Technical Guidelines for Risk Assessment of Contaminated Sites* (HJ 25.3–2019), this site is composed of industrial land and belongs in category I, where the HM risk assessment considers the adult and child CR and HI.

**Fig 9:**
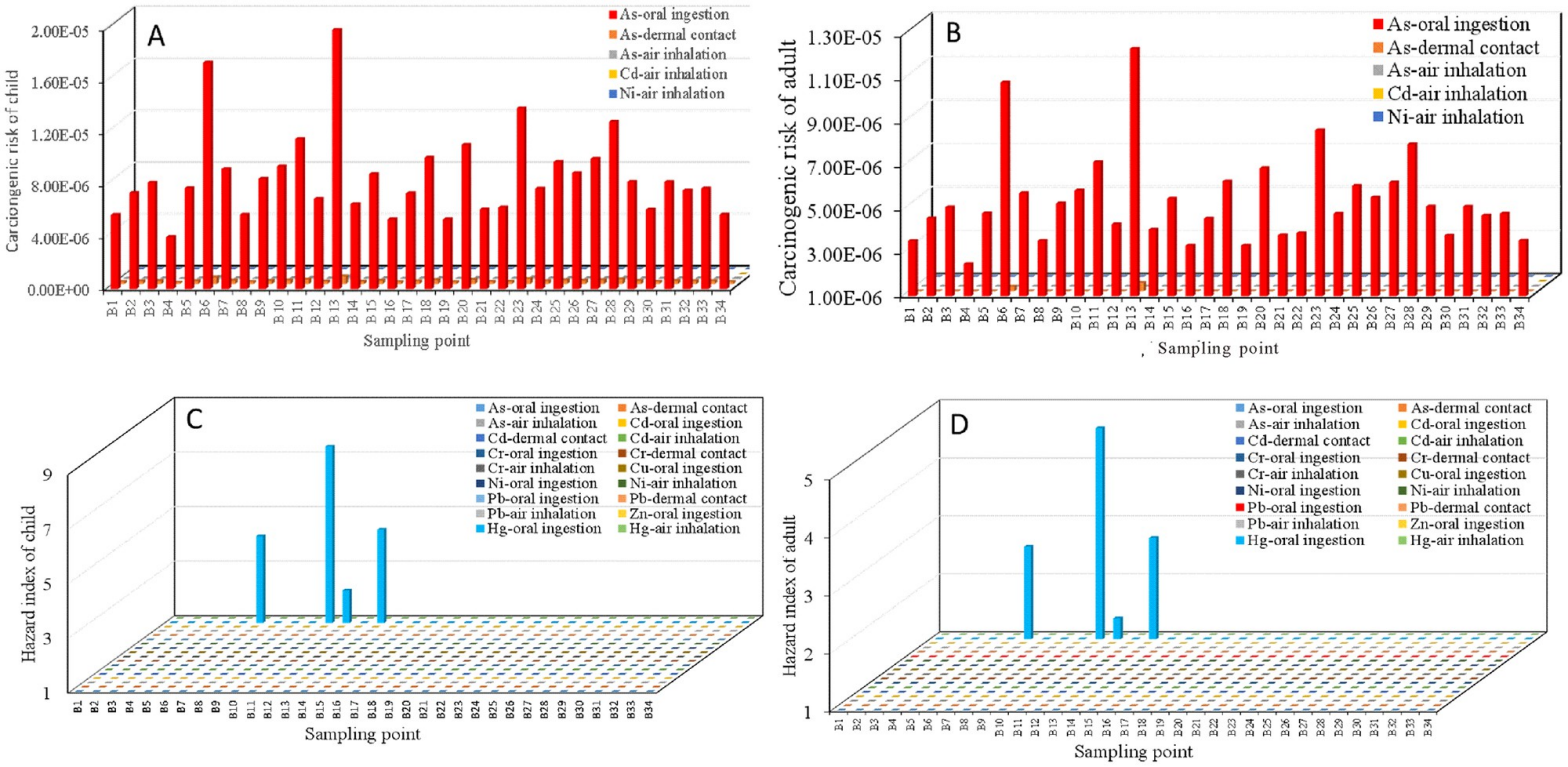
Carcinogenic risk (CR) and hazard index (HI) of heavy metals for three exposure pathways of children and adults. (A) CR-child, (B) CR-adult, (C) HI-child, (D) HI-adult.

Fig 9A and 9B shows the CRs of the three exposure pathways for As, Ni and Cd for children and adults. The CRs for As all exceeded the acceptable level (1×10^−6^) but did not exceed the tolerance value (1×10^−4^); among the pathways, the CR from oral ingestion was highest. The CRs for Cd and Ni did not exceed the acceptable level (1×10^−6^). The CRs for children were generally higher than those for adults, which indicates that children are more vulnerable to soil HMs.

The HM HIs for children and adults are shown in Fig 9C and 9D. Both the child and adult HIs of Hg oral ingestion at B6, B10, B11 and B13 exceeded the tolerance value (>1). The HIs of the other HMs were <1. Similar to the CRs, the HIs were generally higher for children than adults.

#### Source risk assessment

**Fig 10:**
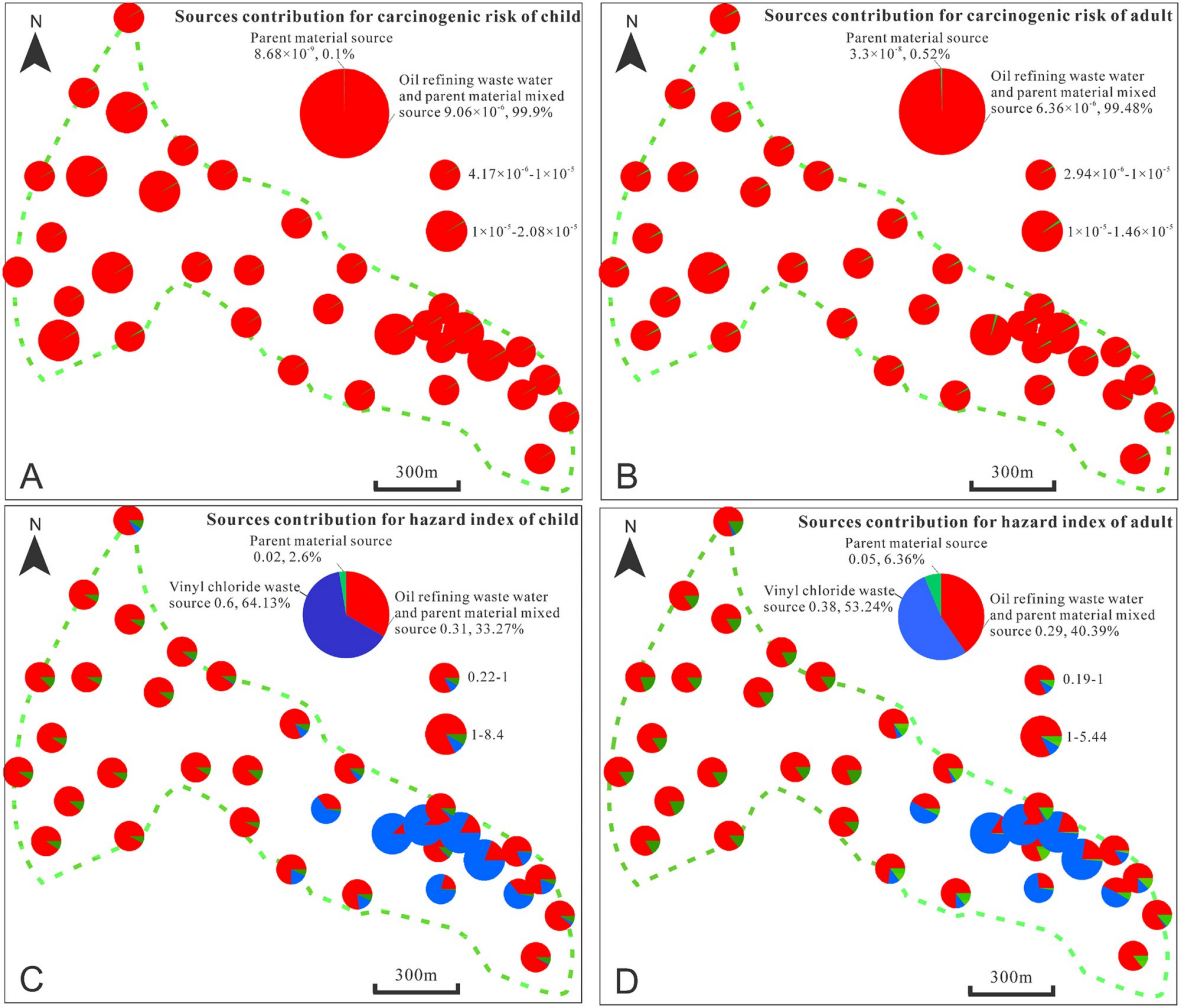
(A) Contribution of heavy metal sources to the total carcinogenic risks for (A) children and (B) adults; (C) contribution of heavy metal sources to the total hazard indices for (C) children and (D) adults.

The CRs for children and adults that showed contamination by As, Cd and Ni pollution sources were analyzed (Fig 10A and 10B). The total CR for children was between 4.17×10^−6^ and 2.08×10^−5^. The CRs at all of the sampling sites were above the acceptable level (1×10^−6^) but did not exceed the tolerance value (1×10^−4^). The mixed oil refining wastewater and parent material source (As) and soil parent material source (Cd and Ni) had average total CRs for children of 9.06×10^−6^ and 8.68×10^−9^, respectively, and contributed 99.9% (mixed source) and 1% (parent material) to the CRs. The same sources had average total CRs for adults of 6.36×10^−6^ (mixed source) and 3.3×10^−8^ (parent material) and contributed 99.48% and 0.52% to the total CRs, respectively. The mixed source (oil refining wastewater and parent material) was the main contributor to the HM CRs at the study site. Both sources increased the CRs in children relative to adults. At the same time, the proportion of the pollution source that dominates the child CR (e.g., oil refining wastewater and parent material mixed source) increases compared to adults.

The HIs for the three pollution sources for children and adults are shown in Fig 10C and 10D. The total HIs for children was between 0.22 and 8.4. There were four samples where the total HI exceeded the tolerance value (>1); the HI was highest at B10 and B13. The total average HIs of all three sources were 0.31 (mixed), 0.6 (vinyl chloride waste source) and 0.02 (parent material) with contributions to the total average HI of 33.27%, 64.13% and 2.6%, respectively. The total HIs for adults were between 0.19 and 5.44. There were four samples where the total HIs exceeded the tolerance value (>1). Similar to the child HIs, they were highest at B10 and B13. The total average HIs of the sources were 0.29 (mixed), 0.38 (vinyl chloride waste source) and 0.05 (parent material), with contributions to the total average HI of 40.39%, 52.34% and 6.36%, respectively. Chlor-alkali solid waste was the main pollution source that affected HM HIs at the site. Similar to the CRs, the proportion of the primary pollution source (chlor-alkali solid waste source) for the HI increases for children compared with adults.

The child and adult CRs did not exceed the tolerance value (1×10^−4^), but the HIs of four samples exceeded that tolerance value (>1). All four samples were recovered from the eastern boundary of the site, which was originally an industrial sewage outlet. Therefore, this area was key for soil remediation at the site. The HM concentrations in the farmland adjacent to the site showed that Hg concentrations exceed local soil background values, indicating that pollution at the site had spread to the farmland.

Compared with deterministic risk assessments, those based on pollution sources could better reflect the sources of pollutants at contaminated sites and the levels of soil pollution. Thus, this information could provide guidance for the next steps in soil remediation and evidence for the management of similar industrial sites [10, 11]. In subsequent research, the forms and bioavailability of HMs in soil should be measured to comprehensively evaluate environmental and ecological hazards. Additionally, HM contents in air, water and other environmental media should be measured. Furthermore, possible HM sources could be analyzed in more detail with isotope tracing. In this study, soil samples were collected and human behavior was estimated empirically. To optimize the risk assessments to human health and improve the accuracy of the assessments, behavioral characteristics of the local population were obtained by questionnaires.

## Conclusions

The level of Hg pollution was most severe in landfills with Hg-containing waste, as well as As, Cu and Pb contamination. Three pollution sources were identified from the studied HMs: oil refinery wastewater and parent material (mixed source); PVC waste; parent material. The different pollution sources contributed differently to the ecological risk, CRs and HIs of the soil.vinyl chloride waste source contributed the highest ecological risk, with Hg demonstrating the most significant impact on the soil ecological risk. The mixed source contributed the highest total average CRs for children and adults while vinyl chloride waste source contributed the highest average total HIs to children and adults. Overall, children’s health was more susceptible to the major sources of pollution.

## Supporting information

S1 Data (XLSX)

S1 Table Reference dose (RfD) and slope factor (S_F_) of heavy metals (HMs). (DOCX)

S2 Table Values of relevant parameters in the human health risk assessments. (DOCX)
