# Supplementary material for: Characteristics, sources and risk assessments of heavy metal pollution in soils of typical chlor-alkali residue storage sites in northeastern China
Source: PLoS One. 2022 Sep 9;17(9):e0273434. doi: 10.1371/journal.pone.0273434 (PMC9462793; doi:10.1371/journal.pone.0273434)
Supplement: S1 Table — (DOCX) [file pone.0273434.s002.docx]

**S1 Table. Reference dose (RfD) and slope factor (S_F_) of heavy metals (HMs)**

| HMs | RfD/mg·(kg·d)^-1^ | | | S_F_/kg·d·mg ^-1^ | | |
| --- | --- | --- | --- | --- | --- | --- |
|  | Oral ingestion (RfD_o_) | Dermal contact (RfD_d_) | Air inhalation (RfD_i_) | Oral ingestion (*S*_Fo_) | Dermal contact (*S*_Fd_) | Air inhalation (*S*_Fi_) |
| As | 3.00E−04 | 3.00E−04 | 3.00E−04 | 1.50E+00 | 3.66E+00 | 1.50E+01 |
| Cd | 1.00E−03 | 2.50E−05 | 5.71 E−05 | — | — | 6.30E+00 |
| Cr | 1.5 E+00 | 1.95E−02 | 2.86E−05 | — | — | — |
| Cu | 4.00E−02 | 4.00E−02 | — | — | — | — |
| Hg | 1.60E−04 | 1.60E−04 | 8.75E−05 | — | — | — |
| Zn | 3.00E−01 | 3.00E−01 | 3.00E−01 | — | — | — |
| Pb | 1.40E−04 | 1.40E−04 | — | — | — | — |
| Ni | 2.00E−02 | 8.00E−04 | 2.06E−02 | 1.70E+00 | 4.25E+01 | 9.01E−01 |

Note: — indicates no data
