# Supplementary material for: Characteristics, sources and risk assessments of heavy metal pollution in soils of typical chlor-alkali residue storage sites in northeastern China
Source: PLoS One. 2022 Sep 9;17(9):e0273434. doi: 10.1371/journal.pone.0273434 (PMC9462793; doi:10.1371/journal.pone.0273434)
Supplement: S2 Table — (DOCX) [file pone.0273434.s003.docx]

**S2 Table. Values of relevant parameters in the human health risk assessments**

| Parameters | Value | Unit |
| --- | --- | --- |
| OSIR_a_ | 100 | mg·d^-1^ |
| OSIR_c_ | 200 | mg·d^-1^ |
| ED_a_ | 24 | a |
| ED_c_ | 6 | a |
| EF_a_ | 350 | d·a^-1^ |
| EF_c_ | 350 | d·a^-1^ |
| BW_a_ | 65 | kg |
| BW_c_ | 19.2 | kg |
| ABS_o_ | 1 | D |
| AT_ca_ | 27740 | d |
| AT_nc_ | 2190 | d |
| SAF | 1 | D |
| SSAR_a_ | 0.07 | mg·cm^-2^ |
| SSAR_c_ | 0.2 | mg·cm^-2^ |
| E_v_ | 1 | time^-1^ |
| H_a_ | 161.5 | cm |
| H_c_ | 113.15 | cm |
| SER_a_ | 0.32 | D |
| SER_c_ | 0.36 | D |
| PM_10_ | 0.074 | mg·m^-3^ |
| DAIR_a_ | 16.3 | m^3^·d^-1^ |
| DAIR_c_ | 7.5 | m^3^·d^-1^ |
| PIAF | 0.75 | D |
| fspi | 0.8 | D |
| fspo | 0.5 | D |
| EFI_a_ | 262.5 | d·a^-1^ |
| EFI_c_ | 262.5 | d·a^-1^ |
| EFO_a_ | 87.5 | d·a^-1^ |
| EFO_c_ | 87.5 | d·a^-1^ |
| PM_10_ | 0.119 | mg.m^-3^ |

Note: Units demarcated with D are dimensionless
